# Identification of a male-produced sex-aggregation pheromone for a highly invasive cerambycid beetle, *Aromia bungii*

**DOI:** 10.1038/s41598-017-07520-1

**Published:** 2017-08-04

**Authors:** Tian Xu, Hiroe Yasui, Stephen A. Teale, Nao Fujiwara-Tsujii, Jacob D. Wickham, Midori Fukaya, Laura Hansen, Satoshi Kiriyama, Dejun Hao, Akio Nakano, Longwa Zhang, Takahito Watanabe, Masahiko Tokoro, Jocelyn G. Millar

**Affiliations:** 1 College of Environmental Science and Forestry, State University of New York, Syracuse, NY 13210 USA; 2 Laboratory of Chemical Ecology, Central Region Agricultural Research Center, NARO, Tsukuba, Ibaraki 305–8666 Japan; 3 Institute of Zoology, Chinese Academy of Sciences, Beijing, China; 4 Department of Entomology, Rutgers University, Rutgers New Jersey, USA; 5 College of Bioresource Sciences, Nihon University (NUBS), Fujisawa, Kanagawa 252-0880 Japan; 6 Department of Forest Protection, Nanjing Forestry University, Nanjing, China; 7 Tokushima Agriculture, Forestry, and Fisheries Technology Support Center, Myozai, Tokushima 779-3233 Japan; 8 Anhui Provincial Key Laboratory of Microbial Control, School of Forestry & Landscape Architecture, Anhui Agricultural University, Anhui, Hefei 230036 China; 9 Forestry & Forest Products Research Institute, Tsukuba, Ibaraki 305-8687 Japan; 10 Departments of Entomology and Chemistry, University of California, Riverside, CA 92521 USA

## Abstract

The longhorned beetle *Aromia bungii* (Coleoptera: Cerambycidae) is a major pest of stone fruit trees in the genus *Prunus*, including cherries, apricots, and peaches. Its native range includes China, Korea, Mongolia, and eastern Russia, but it has recently invaded and become established in several countries in Europe, and Japan, and it has been intercepted in shipments coming into North America and Australia. Here, we report the identification of its male-produced aggregation pheromone as the novel compound (*E*)-2-*cis*-6,7-epoxynonenal. In field trials in its native range in China, and in recently invaded areas of Japan, the pheromone attracted both sexes of the beetle. Thus, the pheromone should find immediate use in worldwide quarantine surveillance efforts to detect the beetle in incoming shipments. The pheromone will also be a crucial tool in ongoing efforts to eradicate the beetle from regions of the world that it has already invaded.

## Introduction

**Figure 1:**
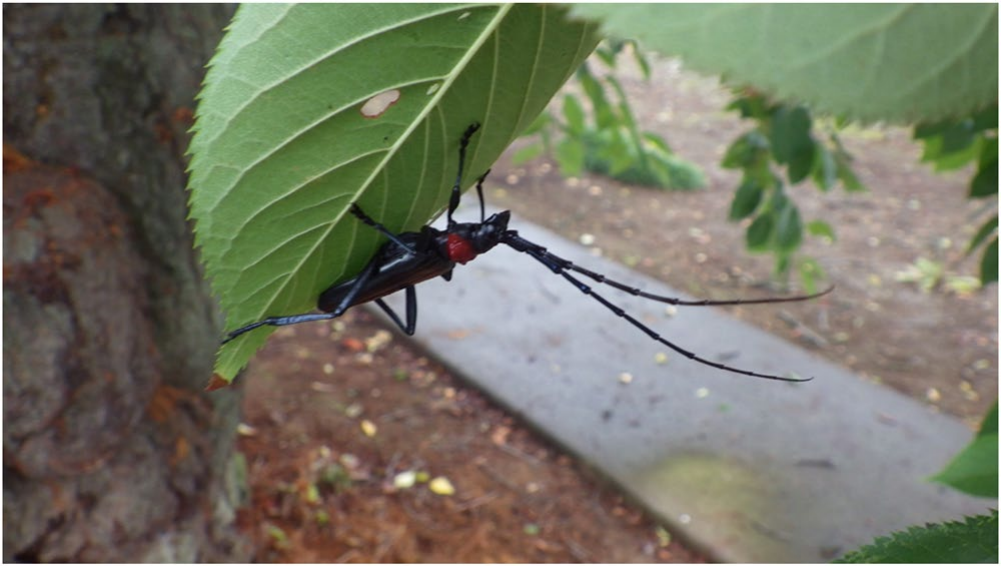
Male *Aromia bungii* on a cherry leaf (Gunma Prefecture, Japan, July 2016).

The invasive wood-boring beetle *Aromia bungii* (Faldermann) (Coleoptera:Cerambycidae, subfamily Cerambycinae, tribe Callichromatini) (Fig. 1) infests trees in the genus *Prunus*, which includes a number of economically important stone fruit trees such as peaches, plums, cherries, and apricots^1, 2^. It is one of only four species in the genus worldwide^1^. As with many cerambycid beetles, the developing larvae feed in the nutrient-rich phloem, cambium, and outer sapwood, and overwinter as larvae, sometimes for several sequential years. The mature larvae bore into the xylem to form a pupation chamber, from which the diurnally active adults emerge in midsummer. Feeding damage to the vascular tissues and the weakening of the trunk and branches by larval tunneling frequently kills the host tree. Because the developing larvae are hidden within the subcortical tissues of their hosts, the beetle is difficult to control with insecticides.

**Figure 2:**
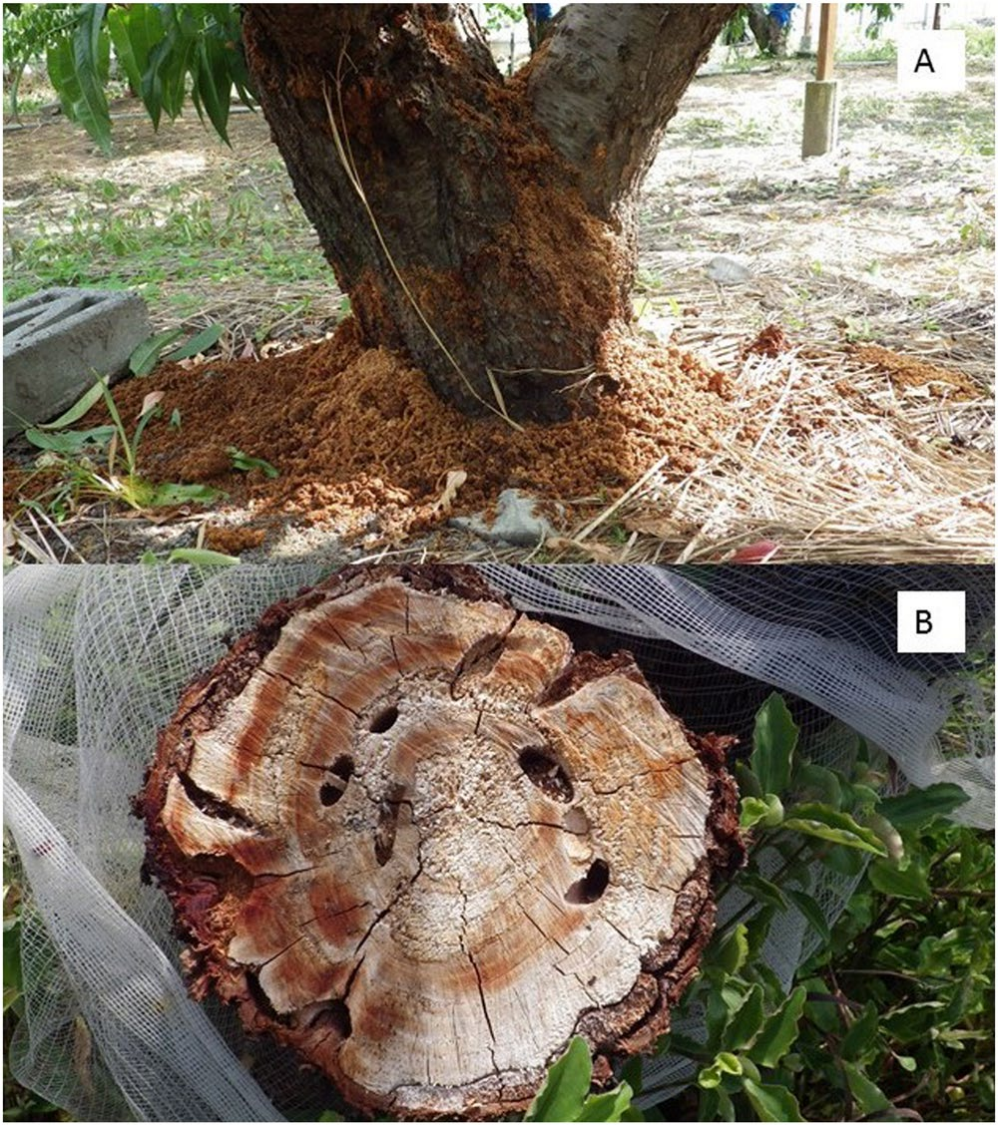
(**A**) A peach tree infested by *A. bungii* (Tokushima Prefecture, Japan, August 2016), showing the voluminous frass produced by the feeding larvae; (**B**) structural damage to the trunk caused by developing larvae feeding in the nutrient-rich phloem and tunneling in the heartwood.

*Aromia bungii* is native to China, Korea, Mongolia, and eastern Russia, where its life cycle is reported to be from 2–4 years, depending on climate^3, 4^. It recently has invaded and become established in Japan and several countries in Europe, including Italy^5^ and Germany^6^. It also has been intercepted in the United Kingdom, the United States, and Australia^7^. Although it is not yet reported to have established in the latter three countries, it is considered an economically dangerous invasive species, with surveillance and quarantine measures in place in all three countries to exclude it. Its potential danger to stone fruits is dramatically highlighted by its recent invasion of Japan. Since first being reported in Japan in 2013^8^, it already has caused substantial damage to peach orchards and Japan’s iconic cherry blossom trees (Fig. 2).

Current quarantine surveillance methods for the beetle are limited to visual inspections of host plant materials and wooden products, which are of limited efficacy in detecting larvae deep within wood^1^. In the field, infestations can be detected in their later stages by the accumulation of larval frass and exit holes in the tree trunks, and for heavy infestations, visual observation of the large, colourful, and diurnally active adults (Fig. 1). Use of fermenting liquids as trap baits has been reported^9^, but the efficacy of these baits is unclear. Because of its recent invasion of Japan and Europe, its likely invasion of additional countries, and its economic importance, including in its native range, sensitive methods of sampling this species are urgently needed, both for detecting new infestations, and for use in ongoing control and eradication efforts in areas in which it has established.

As part of an ongoing program to elucidate the chemical ecology of cerambycid beetles, we report here the identification of the male-produced sex-aggregation pheromone (*sensu* Cardé)^10^ of *A. bungii*. The identification was carried out simultaneously and independently by a Japanese team, and a joint Chinese and American team. The synthesized pheromone was field-tested in China in the beetles’ native range, and in Japan in a newly-infested region where the beetle is already causing substantial damage. The pheromone was attractive to beetles of both sexes, and so should provide a valuable tool for use in ongoing worldwide efforts to detect, contain, and eradicate this economically important invasive species.

## Results

### Identification of the male-produced pheromone

**Figure 3:**
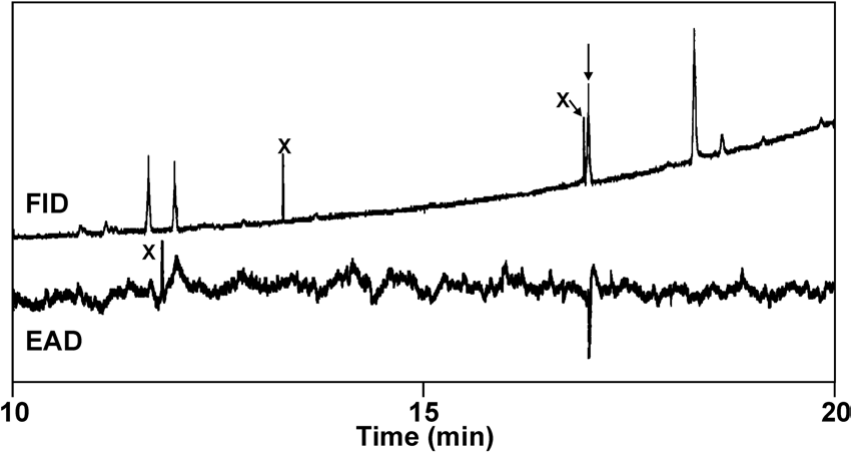
Analysis by coupled gas chromatography-electroantennogram detection of an extract of headspace volatiles produced by male *Aromia bungii*. Top trace shows the GC detector response; bottom, inverted trace shows the corresponding response from the antenna of a female *A. bungii*. Peaks marked with an X are artefacts.

Extracts of headspace volatiles collected from male and female *A. bungii* in Japan were analyzed by coupled gas chromatography-electroantennogram detection (GC-EAD, Fig. 3), which showed that some extracts from males contained a sex-specific compound which elicited strong responses from beetle antennae. The compound had a Kovat’s index of 2016 on the polar INNOWax column used in these analyses. Further analyses by coupled gas chromatography-mass spectrometry (GC-MS) determined that the compound had a Kovat’s index of 1332 on a lower polarity DB-5 GC column. In addition, extracts of males contained small amounts of (2*E*,6Z)-nona-2,6-dienal (9.8 ± 7.3% of major compound, n = 3), and extracts from both sexes contained small amounts of benzyl alcohol and benzaldehyde. The major male-produced compound was detected in six of the eight extracts prepared in Japan by rinsing jars in which beetles had been held. The compound also was detected in all 12 of the extracts of male beetle headspace odours prepared in Japan, in amounts estimated at ~250 ng to 10 µg per extract, and in eight of the 23 aeration extracts prepared in China. The compound was not detected in any of the extracts prepared from female beetles in either country.

**Figure 4:**
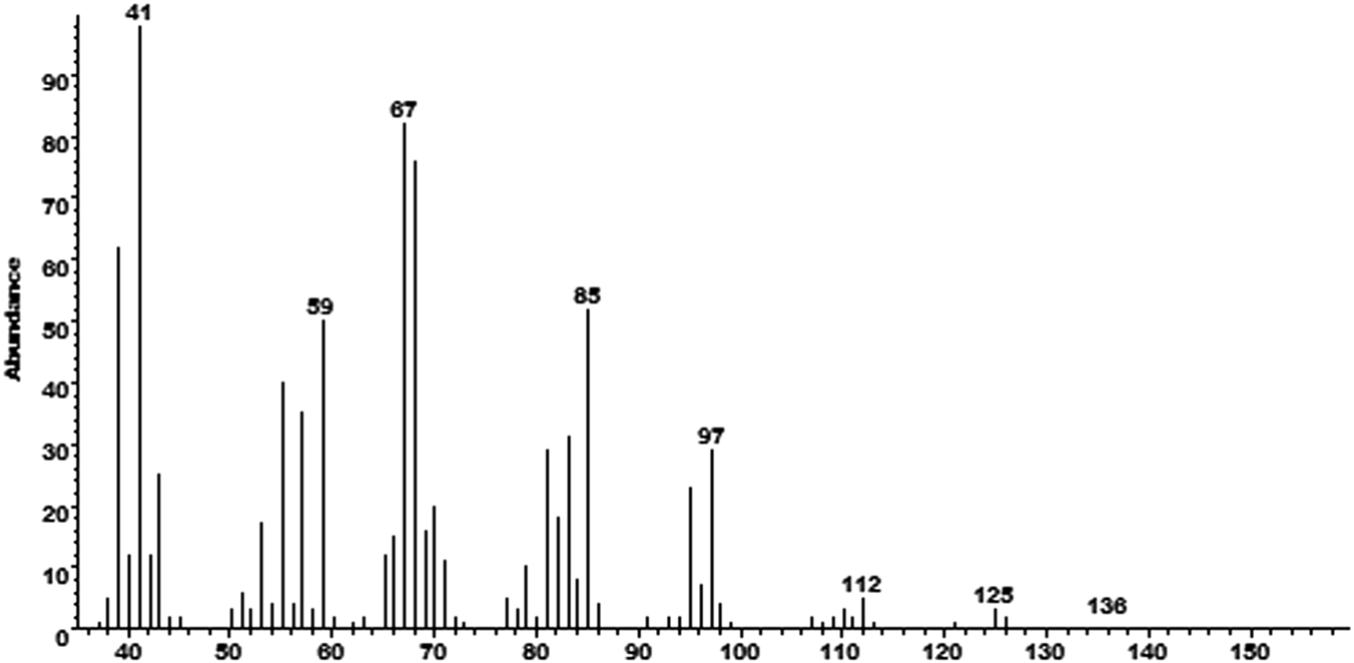
Electron impact ionization mass spectrum of the compound produced sex-specifically by male *Aromia bungii* that elicited responses from antennae of females.

The electron impact ionization mass spectrum of the major compound detected in extracts from both Japan and China was minimally informative (Fig. 4), but suggested a molecular weight of 154 daltons based on a trace ion visible in some analyses, and a slightly larger ion at *m/z* 136 corresponding to loss of water from the molecular ion. Hydrogenation over a palladium catalyst resulted in the disappearance of the parent compound, suggesting that there was at least one C = C double bond. However, none of the products from hydrogenation could be identified. In addition, during removal of the catalyst by filtration of the hydrogenation products through a pad of celite filtering aid, eluting with pentane, the reduction products remained adsorbed on the celite, indicating that they were of at least moderate polarity, i.e., that they contained one or more oxygens or other heteroatoms.

The proton NMR spectrum obtained on ~10 µg of material purified by preparative GC was much more informative. Thus, a one-proton doublet at 9.51 ppm with a large coupling constant (J = 7.8 Hz) was assigned to an aldehyde conjugated to a *trans*-1,2-disubstituted alkene, with the alkene protons appearing at 6.13 (ddt, 1 H, J = 15.6, 7.8, 1.5 ppm) and 6.90 ppm (dt, 1 H, J = 15.6, 6.8 Hz). The chemical shifts and coupling patterns of two single-proton multiplets at 2.92 (apparent td, 1 H, J = 7.3, 4.7 Hz) and 2.88 ppm (apparent td, 1 H, J = 6.5, 4.7 Hz) suggested an epoxide flanked by methylene groups with diastereotopic protons at 1.76 and 1.67 ppm (both m, 1 H) and 1.52 ppm (m, 2 H). The protons at 1.76 and 1.67 ppm were coupled to an allylic methylene at 2.51 ppm (m, 2 H), whereas the methylene at 1.52 ppm was coupled to a methyl group at 1.03 ppm (t, 3 H, J = 7.5 Hz). ^1^H-^1^H COSY confirmed the connectivity, and suggested that the structure was the monoepoxide of (2*E*,6*Z*)-nona-2,6-dienal. The magnitude of the coupling constant between the two epoxide protons (4.7 Hz) supported the assignment of the epoxide as *cis* rather than *trans*. The identification was confirmed by synthesis of the monoepoxides of both (2*E*,6*Z*)-nona-2,6-dienal and (2*E*,6*E*)-nona-2,6-dienal; the proton NMR spectrum of the former was a close match with that of the insect-produced compound, whereas the epoxide and methyl protons were shifted significantly upfield to 2.67 and 0.96 ppm respectively in the spectrum of (*E*)-2-*trans*-6,7-epoxynonenal. Furthermore, the mass spectra of the synthetic compound and the insect-produced compound, and their retention times/retention indices on both the polar INNOWax and nonpolar DB-5 columns, matched exactly.

### Field bioassays in China

**Figure 5:**
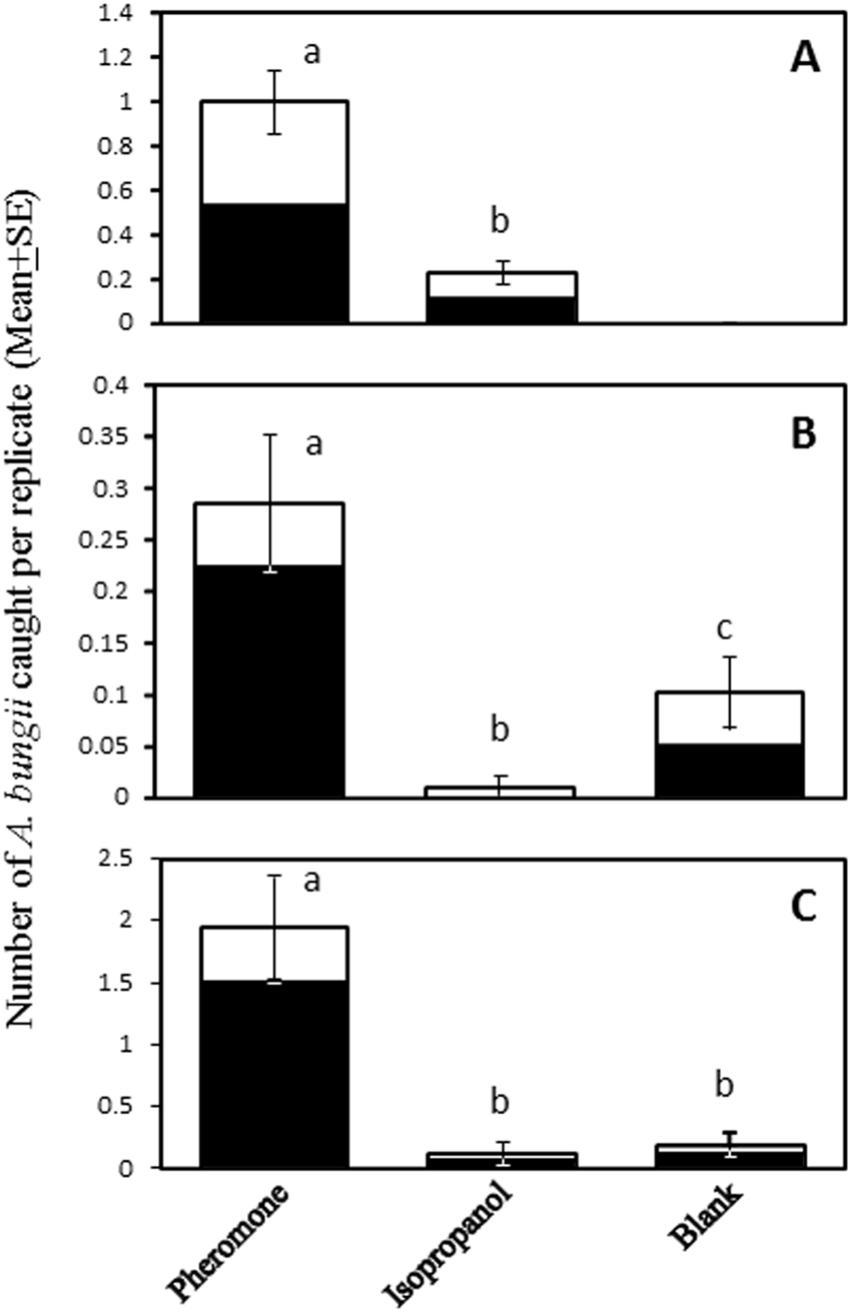
Numbers of *A. bungii* captured in traps baited with (**A**) the putative pheromone [(*E*)-2-*cis*-6,7-epoxynonenal, 25 mg] and control (isopropanol) in the first bioassay in China; and (**B**) and **(C**) (*E*)-2-*cis*-6,7-epoxynonenal, isopropanol, and unbaited traps in the second bioassay in China and the bioassay in Japan, respectively. White bars represent males and black bars represent females. Bars with the same letter are not significantly different (P > 0.05). Each pair of treatments was compared using Poisson regression analysis followed by pairwise contrasts.

A total of 59 male and 69 female *A. bungii* were caught in the first field trial in China (Fig. 5A). Significantly more *A. bungii* were caught in traps baited with (*E*)-2-*cis*-6,7-epoxynonenal lures than those with isopropanol control lures (P < 0.0001). When males and females were analyzed separately, both sexes were significantly attracted to the pheromone lures (Fig. 5A; males, P < 0.0001; females, P < 0.0001). There was no significant difference between the sex ratios of beetles caught in traps baited with pheromone (*X* ^2^ = 0.36, P = 0.55) or the control (*X* ^2^ = 0.043, P = 0.83).

In the second bioassay (Fig. 5B), a total of 47 *A.bungii* were captured (18 males and 29 females). Pheromone-baited traps again caught significantly more beetles of both sexes combined than either the isopropanol solvent (P < 0.0001) or completely blank control (P = 0.0003) treatments. Examining the sexes separately, traps with pheromone and blank traps captured significantly more males than those with isopropanol (P = 0.0034 and 0.012, respectively), suggesting that isopropanol might be inhibitory to males. Significantly more females were captured by traps with pheromone lures compared to either traps with isopropanol (P < 0.0001) or blank traps (P < 0.0001). Traps with pheromone caught significantly more females than males (*X* ^2^ = 9.13, P = 0.0025), while there was no significant sex bias among the beetles caught in either isopropanol or blank traps (*X* ^2^ = 2.78, P = 0.096; X^2^ = 0, P = 1.000, respectively).

### Field bioassays in Japan

A total of 9 males and 27 females were trapped (Fig. 5C). Pheromone baited traps captured significantly more *A. bungii* (sexes combined) than either the isopropanol solvent (P = 0.0005) or the completely blank control (P = 0.0003). When the sexes were analyzed separately, marginally more male beetles were caught in traps with pheromone than traps with either isopropanol or blank traps (Fig. 5C; P = 0.056 for both). Significantly more females were caught in traps with pheromone than traps with either isopropanol or blank traps (P = 0.0032 and 0.0012, respectively). Significantly more females than males were caught by pheromone-baited traps (*X* ^2^ = 9.32, P = 0.0023). Overall, these results indicated attraction of both sexes of *A. bungii* to (*E*)-2-*cis*-6,7-epoxynonenal, but with a female bias.

## Discussion

The analytical and bioassay data presented above provide strong evidence that (*E*)-2-*cis*-6,7-epoxynonenal is the major component of the male-produced sex-aggregation pheromone of *A. bungii*. In GC-EAD analyses, antennae of female beetles responded strongly and specifically only to this compound, even though some extracts contained several other compounds in roughly equal proportions. Similarly, in field bioassays, beetles of both sexes were significantly more attracted to pheromone-baited traps than to control traps in field trials conducted in the beetle’s native region, and a country which it has recently invaded. It also must be emphasized that in the field trial in Japan, to minimize further infestation, all visible beetles were being collected by hand daily throughout the trial. Thus, trap catches would likely have been much higher in the absence of these control efforts.

The structure of the pheromone component, (*E*)-2-*cis*-6,7-epoxynonenal, is unlike that of any previously reported cerambycid pheromones^11^, and is the first pheromone reported from the tribe Callichromatini. Other *Aromia* species, such as *A. moschata* (L.) are commonly known as musk beetles because of the scents which they produce when threatened. The main compounds in the secretions of *A. moschata* were identified as isomers of rose oxide and iridodial^12^, but it is not known whether these compounds are strictly defensive, or whether they may also have roles as pheromones. Furthermore, these oxygenated monoterpenoid structures are clearly products of a different biosynthetic pathway than the unbranched (*E*)-2-*cis*-6,7-epoxynonenal, which could conceivably be produced by oxidative cleavage of an omega-3 polyunsaturated fatty acid such as linolenic acid to give (3*Z*,6*Z*)-nona-3,6-dienal, followed by isomerization to the thermodynamically more stable (2*E*,6Z)-nona-2,6-dienal and epoxidation of the unconjugated double bond.

The effectiveness of the lures and the trapping system can almost certainly be improved by optimizing operational factors such as the dose/release rate of the lures and their effective field lifetimes, and possible additive or synergistic effects of (2*E*,6Z)-nona-2,6-dienal, the minor component released only by males. It also remains to be determined which enantiomer of (*E*)-2-*cis*-6,7-epoxynonenal is produced, and whether the “unnatural” enantiomer causes any inhibition of attraction, although this seems unlikely given the significant attraction to the racemic compound. Nevertheless, because the beetle is currently invading Japan and several countries in Europe, we deemed it important to reveal the identity of the major component of the pheromone immediately, so that it can be incorporated into ongoing efforts to detect, manage, and hopefully eradicate this pest in the countries which it has recently invaded. Various aspects of trap and lure optimization are the subject of ongoing work.

## Materials and Methods

### Collection of insect-produced volatiles in China

Adult *A. bungii* were hand collected on the campus of the Nanjing Forestry University (32°04′46.95″N 118°48′47.40″E) in Nanjing, Jiangsu Province, China in 2014 and 2015, and stored in plastic cups until used in experiments. To collect headspace odours, charcoal-filtered air was pulled (30–500 ml/min) through stoppered 2 L glass flasks containing individual beetles for 24 h by portable vacuum pumps (Airlight, SKC Inc., Eighty-Four, PA, USA). The air outlets were fitted with volatiles traps made of Porapak Q adsorbent (200 mg; Sigma-Aldrich, St. Louis, MO, USA) secured in glass tubes by glass wool plugs. The Porapak Q was initially cleaned by Soxhlet extraction with dichloromethane, and traps were rinsed with dichloromethane before each use. Trapped volatiles were eluted from traps with dichloromethane (0.5 ml). In total, 23 aeration extracts were prepared from male beetles, and 7 extracts from females.

### Collection of insect-produced volatiles in Japan

No official permits were required for collection of insects in Japan. Adult *A. bungii* were hand collected at Soka City (35°49′30.3″N 139°48′27.9″E), Saitama Prefecture, Japan during late June to July, 2015, soon after their emergence from cherry blossom trees, “Somei-yoshino” [Cerasus × yedoensis (Matsum.) A. V. Vassil], which had been netted at the base for collection. Each adult was kept individually in a plastic cup and provided a cotton ball soaked with diluted sucrose water under conditions of 15 °C and 15 h/9 h light/dark (lights on 05:00, off 20:00) lighting conditions. Single beetles were held in 100 ml glass beakers covered with aluminum foil and kept 2 h at 25 °C. After removal of the beetles, the beakers were rinsed with 1 ml of *n*-hexane. The hexane extracts were kept at −30 °C until analyzed. In total, eight extracts were prepared from males, and two from females. Twelve aeration extracts also were prepared from single males or pairs of males held in 1 L glass flasks, collecting volatiles on Porapak Q adsorbent similar to methods described above.

### Analysis of extracts by coupled gas chromatography-electroantennogram detection (GC-EAD)

The extracts of beetle volatiles were analyzed by GC-EAD using a Hewlett-Packard 5890 series II GC fitted with a HP-INNOWax column (30 m × 0.32 mm ID × 0.25 μm film thickness, Agilent Technologies, Santa Clara, CA, USA). The injector and detector temperatures were 250 °C, and injections were made in splitless mode. The oven was programmed from 50 °C for 1 min, then 10 °C per min to 250 °C, using helium carrier gas at a linear velocity of 50 cm/sec. The FID detector temperature was 250 °C. Helium makeup gas (10 ml per min) was added to the column effluent via a stainless steel T-union, after which the flow was split equally between the GC’s flame ionization detector (FID) and the EAD with a press-fit Y splitter (Agilent). The GC effluent for EAD was directed to a glass transfer tube (15 mm ID) mounted on the GC and was mixed with humidified air (300 ml per min, 20 °C), and then passed over the antennal preparation. An antenna including the basal segment was gently removed from a live beetle with scissors and forceps, and was mounted on a Syntech EAG probe (Syntech, Kirchzarten, Germany), making connections with electrode gel (Spectra® 360, Parker Lab. Inc., NJ, USA). EAG and FID signals were fed into a computer through an analogue-to-digital conversion board (IDAC-232, Syntech), and the signals were displayed and analyzed with Syntech GC–EAD software. In total, analyses were replicated with antennae from four females and two males, with each antennal preparation being reused for 2–3 analyses.

### Identification of insect-produced compounds

Extracts of insects from China were analyzed on an Agilent 78020 A GC interfaced to an Agilent 5977E mass selective detector (MSD, Agilent). The GC was fitted with a low polarity DB-5MS column (30 m × 0.25 mm ID × 0.25 μm film; J&W Scientific, Folsom CA, USA), and the oven temperature was programmed from 40 °C for 1 min, then 10 °C per min to 280 °C, hold for 10 min, with helium carrier gas. The injector temperature was 250 °C, and the transfer line 280 °C. Injections were made in splitless mode. Mass spectra were taken in EI mode (70 eV). Extracts of insects from Japan also were analyzed by GC-MS on a polar INNOWax column, using oven temperature conditions as described above. Some compounds were tentatively identified by mass spectral matches with database spectra, with identifications then confirmed by matching retention times and mass spectra of the insect-produced compounds with those of authentic standards, obtained as follows: benzyl alcohol, benzaldehyde, (2*E*,6*E*)-nona-2,6-dienal all from Aldrich Chemical (Milwaukee WI, USA), and (2*E*,6*Z*)-nona-2,6-dienal (Bedoukian Research, Danbury CT, USA). To calculate Kovat’s indices, a set of linear alkanes from C_8_-C_28_ was analyzed under the same conditions.

An aliquot of an extract (~50 μl) was hydrogenated to remove all nonaromatic C = C bonds. Thus, the aliquot was diluted with ~100 μl pentane, ~1–2 mg of 5% Pd on carbon was added, and the mixture was stirred under hydrogen atmosphere for 1 h. The mixture was then filtered through a plug of celite, rinsing successively with pentane and dichloromethane, and the filtrates were concentrated and analyzed by GC-MS.

The major male-specific compound was isolated by preparative GC. Thus, the three extracts containing the largest amounts of the main male-specific compound were combined and concentrated to ~10 µl under a gentle stream of nitrogen. The entire sample was then injected onto a Megabore column (DB-5, 25 m × 0.53 mm ID, 5 µm film thickness; J&W Scientific) over ~30 sec, with an injector temperature of 250 °C, and an oven program of 40 °C for 1 min, then 10 °C per min to 250 °C for 10 min. The column effluent was split ~30:1, with the majority going to a heated outlet port (200 °C). A fraction enriched in the major compound was collected in a dry-ice cooled glass capillary, which, after warming to room temp, was rinsed into a conical vial with ~20 µl of deuterated methylene chloride. The resulting solution was transferred to a 1 mm diameter NMR tube for microbore NMR analyses, which were carried out on a Bruker Avance spectrometer at 600 MHz. Spectra were referenced to the signal from residual CDHCl_2_ in the deuterated solvent.

### Synthesis of monoepoxides of (2*E*,6*Z*)- and (2*E*,6*E*)-nona-2,6-dienals

A solution of (2*E*,6*Z*)- nona-2,6-dienal (27.6 g, 200 mmol; Bedoukian Research) in 1 liter of methylene chloride was cooled to 0 °C, and *meta*-chloroperbenzoic acid (MCPBA, ~70% purity, 54.4 g, ~220 mmol; Aldrich Chemical) was added in 5 portions over 30 min. The resulting mixture was stirred overnight at 0°, by which time all the starting material had been consumed. The resulting white slurry was filtered cold with a Buchner funnel to remove the bulk of the precipitated *meta*-chlorobenzoic acid, and the filtrate was concentrated by rotary evaporation. The residue was taken up in 500 ml hexane and filtered again to remove more precipitated excess MCPBA and *meta*-chlorobenzoic acid, and the filtrate was extracted twice with 2 M aqueous NaOH. The resulting colourless hexane layer was washed with water and brine, dried over anhydrous Na_2_SO_4_, and concentrated under reduced pressure. The residue was purified by Kugelrohr distillation (bp~80 °C at 0.3 mm Hg), producing the monoepoxide as a colorless oil (14.7 g, 48%). ^1^H NMR (CD_2_Cl_2_): δ 9.51 (d, 1 H, J = 7.8 Hz), 6.90 (dt, 1 H, J = 15.6, 6.8 Hz), 6.13 (ddt, 1 H, J = 15.6, 7.8, 1.5 ppm), 2.92 (apparent td, 1 H, J = 7.3, 4.7 Hz), 2.88 (apparent td, 1 H, J = 6.5, 4.7 Hz), 2.51 (m, 2 H), 1.76 (m, 1 H), 1.67 (m, 1 H), 1.52 (m, 2 H), 1.03 (t, 3 H, J = 7.5 Hz). EI-MS (*m/z*, abundance): 154 (M+ , trace), 136 (trace), 125 (2), 112 (4), 97 (22), 95 (17), 85 (43), 83 (22), 81 (22), 68 (62), 67 (66), 59 (46), 57 (31), 55 (35), 41 (100).

A sample of the corresponding monoepoxide from (2*E*,6*E*)-nona-2,6-dienal was synthesized using the same conditions but on a 10 mmol scale. ^1^H NMR (CD_2_Cl_2_): δ 9.51 (d, 1 H, J = 7.8 Hz), 6.87 (dt, 1 H, J = 15.6, 6.8 Hz), 6.11 (ddt, 1 H, J = 15.6, 7.8, 1.5 Hz), 2.68 (m, 1 H), 2.65 (m, 1 H), 2.46 (m, 2 H), 1.77 (m, 1 H), 1.68 (m, 1 H), 1.52 (m, 2 H), 0.96 (t, 3 H, J = 7.5 Hz).

### Field Bioassays in China

Field bioassays were carried out in a fruit orchard at Anhui Agricultural University’s Teaching and Demonstration Base (31°55′36.03″N 117°11′52.15″E) in Hefei, Anhui Province, China. Black flight-intercept panel traps (IPM Technologies, Portland, OR, USA) coated with Teflon® PTFE DISP 30 **(**diluted 10-fold before application; DuPont Chemical Co., Wilmington, DE, USA**)** were hung in a grid orientation with alternating treatments and controls, 1–2 m above the ground, and with traps spaced > 10 m apart. The trap collection cups contained a 1:1 solution of automobile antifreeze and water to kill and preserve trapped insects.

In the first bioassay, traps were baited with pheromone (25 mg racemic (*E*)-2-*cis*-6,7-epoxynonenal in 1 mL isopropanol) or control (1 mL isopropanol) lures (N = 20). Lure solutions were deployed in permeable plastic sachets made from heat-sealable polyethylene tubing (7 cm × 4.9 cm, wall thickness 0.05 mm; cat. no. S-1112 Uline, Pleasant Prairie, WI, USA). Traps were deployed on 15 June 2016, and trap catches were counted on June 18, 20, 23, 26, and 29, for a total of 5 temporal replicates.

The second bioassay used pheromone and control lures prepared as in the first experiment, but added a treatment consisting of traps with no lures at all (N = 14). Traps were deployed on 29 June 2016, and *A. bungii* were collected from traps on July 3, 6, 8, 13, 15, 16, and 24 for a total of 7 temporal replicates. Lures were replaced once, on July 15.

### Field bioassays in Japan

Field bioassays were carried out in a peach orchard (ca. 50 m × 23 m) (34°08′55.5″N 134°27′27.5″E) in Itano county, Tokushima prefecture, Japan. Teflon® coated black flight-intercept panel traps (Alpha Scents Inc., Portland OR, USA) were hung in a grid orientation with alternating treatments and controls, 1–2 m above the ground, and with traps spaced > 5 m apart. The trap collection cups contained a 1:10 solution of unscented detergent and water to kill trapped insects.

Traps were baited with pheromone (50 mg racemic (*E*)-2-*cis*-6,7-epoxynonenal in 1 mL isopropanol), solvent controls (1 mL isopropanol) or with no lures at all (N = 4). Lure solutions were deployed in permeable zippered plastic sachets made from polyethylene (10 cm × 7 cm, 0.04 mm wall thickness; Unipack C-4, Seinichi, Tokyo, Japan). Traps were deployed on 5 July to 3 Aug 2016, and trap catches were counted every day. Traps were replaced weekly and lures were renewed simultaneously.

### Statistical analyses

The numbers of male, female, and combined male plus female *A. bungii* collected were modeled using Poisson Regression followed by pairwise contrast tests for assessing differences between treatment pairs. Multiple comparisons were adjusted using Tukey’s range test to control the family-wise error rate. Residual analysis and influence diagnostics were applied to detect potential outliers among the data. Sex ratios were assessed using the Chi-square test. All statistical analyses were carried out using SAS 9.4 software.
